# A case of type 1 multiple endocrine neoplasia with esophageal stricture successfully treated with endoscopic balloon dilation and local steroid injection combined with surgical resection of gastrinomas

**DOI:** 10.1186/s12876-017-0597-6

**Published:** 2017-03-07

**Authors:** Hiroyuki Matsubayashi, Noboru Kawata, Naomi Kakushima, Masaki Tanaka, Kohei Takizawa, Yoshimi Kiyozumi, Yasue Horiuchi, Keiko Sasaki, Teiichi Sugiura, Katsuhiko Uesaka, Hiroyuki Ono

**Affiliations:** 1 Division of Endoscopy, Shizuoka Cancer Center, 1007, Shimonagakubo, Nagaizumi, Suntogun, Shizuoka 411-8777 Japan; 2 Clinic of Genetic Medicine, Shizuoka Cancer Center, 1007, Shimonagakubo, Nagaizumi, Suntogun, Shizuoka 411-8777 Japan; 3 Division of Pathology, Shizuoka Cancer Center, 1007, Shimonagakubo, Nagaizumi, Suntogun, Shizuoka 411-8777 Japan; 4 Division of Hepato-biliary-pancreatic Surgery, Shizuoka Cancer Center, 1007, Shimonagakubo, Nagaizumi, Suntogun, Shizuoka 411-8777 Japan

**Keywords:** Hypergastrinemia, Multiple endocrine neoplasm type 1 (MEN1), Esophageal stricture, Balloon dilation, Steroid, Ulcer

## Abstract

**Background:**

In type 1 multiple endocrine neoplasia (MEN1), esophageal diseases association with excessive gastrin secretion in Zollinger-Ellison syndrome (ZES) sometimes develop. Here, we reported a case of MEN1/ZES, who developed dysphagia due to reflux esophagitis with severe esophageal stricture. Treatment for his esophageal stricture and ZES was discussed.

**Case presentation:**

A 43-year-old man with progressive dysphagia and diarrhea was referred to the teaching hospital. He had a history of recurrent duodenojejunal perforations despite the anti-secretory medication. Blood examinations revealed elevated serum gastrin, calcium, and parathyroid hormone. Upper gastrointestinal endoscopy demonstrated a severe esophageal stricture, multiple gastroduodenal ulcer scars, and a duodenal submucosal tumor. Enhanced computed tomography showed multiple hypervascular tumors within the pancreas and duodenum, suggestive of MEN1. Genetic examination demonstrated a pathogenic *MEN1* mutation. Repetitive endoscopic esophageal dilatation with intralesional corticosteroid injection, coupled with pancreatoduodenectomy were performed to improve the patient’s symptoms and to treat pancreatic tumors. The histology of multiple tumors in the duodenum and pancreas were all consistent with neuroendocrine tumors. His hypergastrinemia subsided and he remained asymptomatic in his gastrointestinal tract after these treatments.

**Conclusion:**

For esophageal stenosis in case of MEN1/ZES, anti-secretory therapy and endoscopic dilatation with corticosteroid injection could be recommended. However, in refractory cases with repetitive and/or severe complications due to high acid secretion, surgical treatment could be considered as an option.

## Background

Type 1 multiple endocrine neoplasia (MEN1) is an autonomic dominant hereditary syndrome that carries a lifetime risk of developing an endocrine neoplasm in ≥95% of cases. Clinical diagnostic criteria require two or more of the following three items: 1) hyperparathyroidism; 2) pancreatic endocrine neoplasm; and 3) pituitary neoplasm. Germline mutation of the *MEN1* gene is recognized in 80–90% of familial cases and in about 65% of sporadic cases [1]. They are sometimes associated with gastrinomas [Zollinger-Ellison syndrome (ZES)] [1], which induce gastric hypersecretion and cause not only gastroduodenal ulcers but also reflux esophagitis [2], in combination with hypercalcemia due to hyperparathyroidism. Heartburn is a typical symptom of reflux esophagitis and is recognized in about 50% of cases of MEN1. Anti-acid therapies, such as proton pump inhibitors (PPI) and H2 receptor antagonists, are effective [2]. MEN1 with ZES also develops dysphagia due to esophageal strictures in a small proportion (9%) [2], and their endoscopic treatment has rarely been reported. This report presents a case of MEN1 with ZES that developed esophageal strictures which were successfully treated with repetitive procedures of endoscopic dilation with local steroid injection combined with duodenectomy and total pancreatectomy.

## Case presentation

A 43-year-old man visited the nearest hospital for examination of his increasing complaints of nausea and diarrhea for 4 years and recent development of dysphagia. He had a history of duodenal ulcer perforation, and underwent omental patching surgery 4 years earlier, followed by oral treatment with a PPI (30 mg/day of lansoprazole). Octreotide analog was not used. Upper gastrointestinal endoscopy (UGE) demonstrated reflux erosive esophagitis with severe esophageal stricture. Plain computed tomography (CT) showed a urinary tract stone and a pancreatic mass. Despite continuous anti-acid therapy, perforation of the small intestine developed, and he underwent closure surgery. Using the selective arterial secretagogue injection (SASI) test, a response by calcium injection was obtained when examined from the superior mesenteric artery, which connects to the feeding arteries to either the pancreatic head, body, or tail. However, it was not obtained by the examinations from the gastroduodenal artery and splenic artery.

**Fig. 1:**
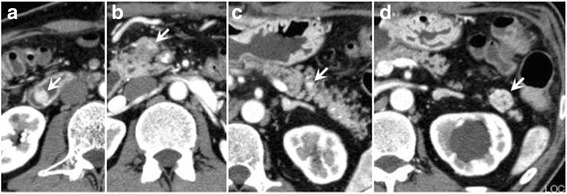
Enhanced computed tomography showing multiple highly vascular tumors within the pancreas and the duodenum; a well demarcated duodenal tumor protruding into the lumen (**a**), a tumor at the head (**b**), body (**c**), and tail (**d**) of the pancreas

**Fig. 2:**
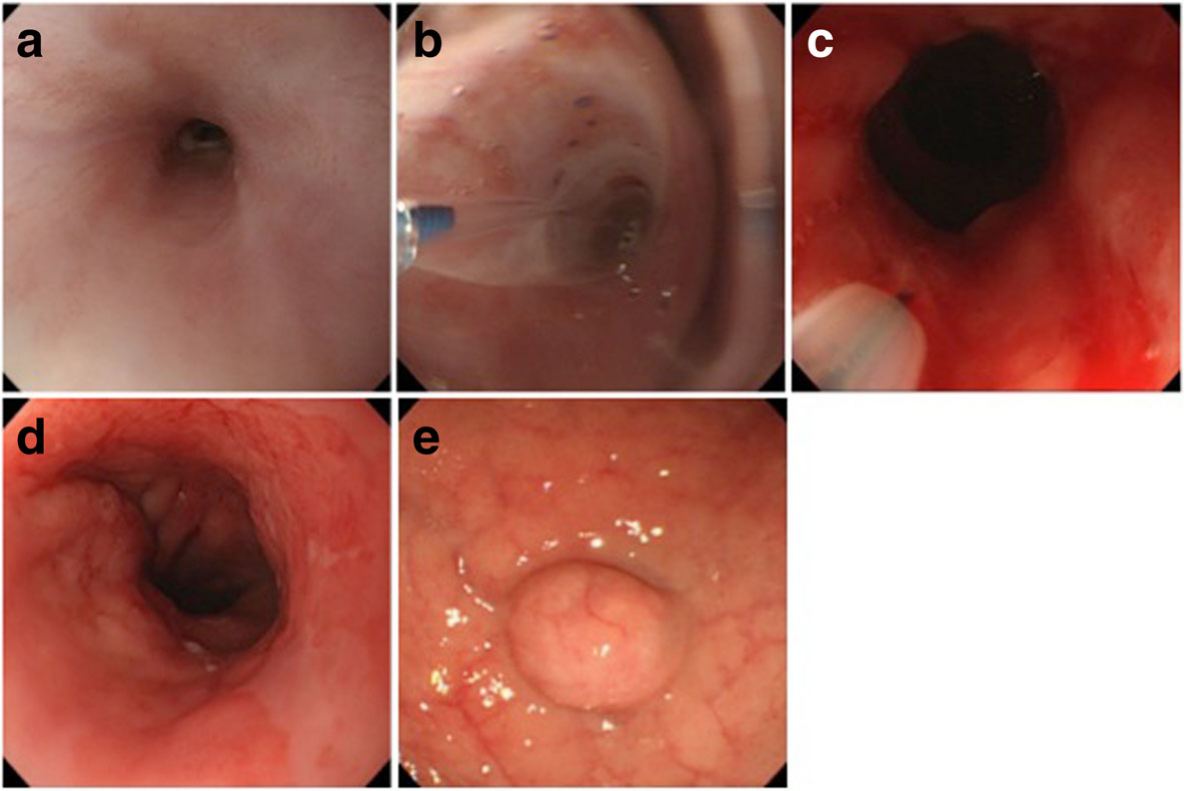
Endoscopic view of the esophageal stenotic portion (**a**), dilation with a balloon catheter (**b**), corticosteroid injection by a needle (**c**), at the last observation (**d**), and the duodenal submucosal tumor (**e**)

The patient was referred to the study hospital to further investigate the suspected diagnosis of MEN1. Blood examination revealed an elevated level of serum gastrin (≥3000 pg/mL, normal: ≤200 pg/mL), glucagon (253 pg/mL, normal: 70–174 pg/mL), calcium (10.9 mg/dL, normal: 8.5–10.2 mg/dL) and intact-parathyroid hormone (PTH) (104 pg/mL, normal: 10–65 pg/mL). Enhanced CT demonstrated multiple highly vascular lesions within the pancreas (head to tail) and duodenum, with up to 20 mm in the pancreas (Fig. 1). UGE demonstrated healing of the esophageal erosion, however the stenosis at the lower esophagus, approximately 4 mm in diameter and 5 cm in length, became so severe that only a slim endoscope (Olympus GIF-XP260, Tokyo, Japan), but not a standard scope (Olympus GIF-H260), could pass through. Multiple gastroduodenal ulcer scars were observed. In the second portion of the duodenum, a submucosal tumor, 12 mm in size, was also recognized (Fig. 2).

Considering these findings associated with hypergastrinemia, it was planned to treat the esophageal stricture and neuroendocrine tumors prior to the treatment of the hyperparathyroidism. Balloon dilator treatment (CRE^TM^, 15–18 mm, Boston Scientific, Marlborough, MA, U.S.A.) (Fig. 2) was repeated 16 times, before and after the total pancreatectomy with duodenectomy, until the disappearance of the esophageal stricture. The interval between each procedure was almost once a week for the first 10 procedures, except for the month of the surgical period, followed by two weeks for the 11^th^-12^th^ procedures, and one to three months for the 13^th^-16^th^ procedures. Balloon dilation was done for 30–60 s with pressure stepped up as in the following: limited up to 1.5–2 atm for the initial five procedures, 3–4 atm for the next five procedures, and 4–5 atm for the remaining procedures. Since full dilation was not obtained until the 5^th^ procedure, 50 mg (5 ml) of triamcinolone acetonide (Kenacort-A, Bristol-Myers Squibb, New York, U.S.A.) was injected to the stenotic portion at the 6^th^-8^th^ procedure. In detail, 0.5–1.0 ml of triamcinolone acetonide solution was injected into 5–8 sites within the stenosis using an injection needle (NeedleMaster, 26 Gy, Olympus, Tokyo, Japan) each after the balloon dilation. The patient’s symptoms and stricture had disappeared by the 15^th^ procedure, and the 16^th^ dilation was performed at the patient’s request (Fig. 2).

**Fig. 3:**
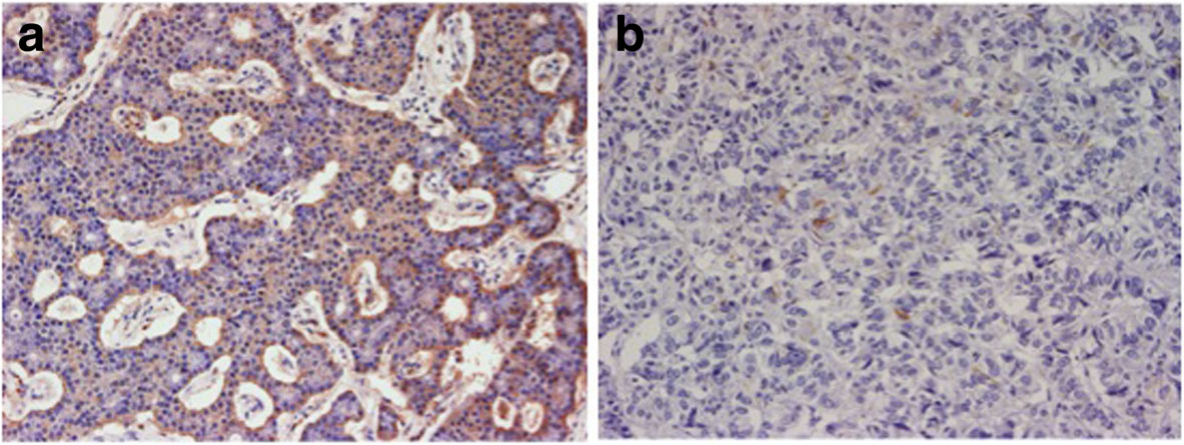
Gastrin expression in the neuroendocrine tumors of the duodenum (**a**) and pancreas (**b**) (Gastrin, x100); nested or diffuse expression in the duodenal tumor (**a**) in contrast to faint expression in the pancreatic tumor (**b**)

Total pancreatectomy with duodenectomy was performed for the multiple masses within the pancreas as well as in the duodenum. Extent of the pancreatectomy was arguable as the result of SASI test was not well informative, to remove possible functional PNET and possible future malignant lesions, total pancreatectomy was performed. This operation was done between the 2^nd^ and 3^rd^ esophageal dilation procedures. The histology of the multiple tumors in the pancreas (4) and duodenum (1) was entirely neuroendocrine tumor (NET), with histological grade 2 (G2). PNETs were recognized in all parts of pancreas (head, body and tail). Immunostaining of gastrin was strongly positive within the duodenal tumor, but only faintly recognized in the pancreatic tumors (Fig. 3). The clinical diagnosis of MEN1 was confirmed with these histological results.

Genetic counseling [3] was completed and his family history was pursued. It was revealed that his father and one of two sisters had hyperparathyroidism, and his father also had a NET with further details unavailable. The patient hoped to have a genetic examination performed after giving informed consent. Germline pathogenic mutation of the *MEN1* gene was detected by whole exon analysis using next generation sequencer (Ion Proton^TM^ system, Thermo Fisher Scientific, Waltham, MA, U.S.A.), which was employed in the on-going institutional project [4], and was confirmed by the commercial base analysis (Falco biosystems, Kyoto, Japan). This information was expected to be used for him and his family in their future medical treatment. To date, his hypergastrinemia improved and he remained asymptomatic after the treatments for 28 months. With taking pancrelipase and insulin injection, his nutrition level and blood glucose level kept in good condition.

## Discussion

This case of MEN1 with pancreaticoduodenal gastrinomas, accompanied with a high level of serum gastrin (≥3000 pg/mL, normal: ≤200 pg/mL), developed severe esophageal stenosis and complained of dysphagia, despite administration of full-dose PPI. He also had repeated histories of duodenojejunal perforations. To treat this condition, repetitive esophageal endoscopic dilations were performed for local therapy and resection of the pancreaticoduodenal NETs for removing the causative lesion of acid hypersecretion.

Benign esophageal stricture can occur in various disorders in adults. For mild to moderate levels of strictures, peptic injury and Schatzki’s ring or web [5] can be causative diseases. Severe strictures can result from caustic ingestion, radiation injury, anastomotic stricture, photodynamic therapy, and/or endoscopic submucosal dissection-related ulcer scar [6–8]. For benign esophageal strictures, endoscopic dilation is usually applied in a stepwise manner depending on its severity [6, 9]. Dilation using a wire-guided polyvinyl dilator (e.g. Savary-Gilliard dilator, COOK, Bloomington, U.S.A.) or balloon dilator (e.g. CRE^TM^) is the first step to treat strictures. Cases with mild to moderate stricture usually need only one to three repetitions of dilation [6, 9] to relieve symptoms. However, cases with severe stricture, especially with long (>2-3 cm) tortuous strictures, or associated with a diameter that precludes passage of a normal diameter endoscope, are defined as complex strictures and often require greater than seven to ten repetitions of dilation procedures and/or additional treatments [6, 9]. In such cases, usually after a few sessions of simple dilation [10], lesional injection of corticosteroid [7, 8, 11] is applied, as was performed in this case. When still refractory to these treatments, incision therapy using needle knife, scissors, and/or argon-plasma coagulation are indicated for anastomotic stricture or Schatzki ring (length <1 cm). In contrast, temporary self-expandable stent placement is applied for strictures after caustic ingestion or radiation injury [6, 9, 10]. In this case, incision was not indicated because of the long stricture and there was no need for temporary stenting as the repetitive balloon dilations with lesional steroid injection were effective. Regarding endoscopic dilation, caution should be paid because adverse events, such as significant hemorrhage (0.3–0.4% [12, 13]), perforation (0.4% [5, 12, 14]), and rarely death (0.4%) [5], can occur. To reduce these complications, so-called “three rules” are generally accepted. Specifically, after moderate resistance is encountered during dilation therapy with Savary-type dilators, no greater than three consecutive dilators should be passed in a single session. As a corollary to this approach, a conservative approach to dilation should be undertaken. For repeat dilation sessions as needed, a luminal diameter of 12 mm or larger is usually adequate to alleviate symptoms of solid dysphagia [9]. As the stricture of the current case was stiff and narrow, careful and repeated dilations were performed until disappearance of the symptoms in a stepwise manner.

In the recently published guideline for the management of PNET [15], surgical exploration for PNET ≤2 cm on imaging studies is still not generally recommended in MEN1/ZES. Even for PNET >2 cm in MEN1/ZES, surgical enucleation is generally recommended, while pancreatoduodenectomy is reserved for specific selected cases. However, in cases of MEN1, multiple small nodules of NET are often recognized within the pancreas and duodenum, as in the current case. In such a case, the choice of surgical resection can be considered if total removal of the tumors must be pursued or preservation of the pancreatic function should be evaluated. Partelli [16] and Sakurai [17] et al. analyzed the biological behavior of non-functioning pancreatic NETs (33 cases and 14 cases, respectively) and reported that those with size ≤20 mm are indolent and of low oncological risk. By contrast, Gibril et al. [18] reported up to 23% of 57 cases of MEN1 with gastrinoma that developed metastasis to the liver following the decision not to perform resection. Gastrinomas with large size (>20 mm) and high gastrin secretion are reported to be aggressive [18]. Imamura et al. [19] recommended surgery based on accurate localization of gastrinomas with the SASI test in MEN1 patients. Hence, a possible surgical option is to remove only functional and aggressive NETs, and leave others untouched. The disadvantages of this strategy are the difficulty of accurate preoperative diagnosis of tumor aggressiveness and of localization by SASI in cases with vascular abnormality or with difficult, super-selective cannulation. In the current case, even a NET in the pancreas tail revealed faint gastrin expression, which may have responded to the SASI test. It is also reported that pancreatic gastrinoma is rare in MEN1, however is more aggressive than duodenal gastrinoma [20]. As most of MEN1 patients are still young when they develop NETs, it is not certain that an indolent NET with small size will maintain its benign nature for its lifespan. Moreover, recent advances in the control of glucose homeostasis after pancreatic resection is improving [21]. Hence, in this case with severe symptoms and repetitive peptic ulcer events, resection, including total pancreatectomy, might be accepted as appropriate care. Future improvement is expected in the evaluation of tumor aggressiveness, variation of the operation, and control of the blood glucose level.

The patient in this case was first suspected of having MEN1 based on the clinical findings of multiple peptic lesions accompanied by high levels of serum gastrin, calcium, and intact PTH, pancreatic and duodenal tumors suspected for NETs, and a urinary tract stone. However, it could not be confirmed until surgery and genetic testing. Confirmation of a germline *MEN1* mutation confirmed this genetic syndrome. This information was beneficial not only for the patient but also for his family [3], especially for the clinical follow up of an affected sister. Actually, the sister began to undergo clinical surveillance of MEN1 with this opportunity, which may allow for early detection of developing a neoplasm in her future.

## Conclusions

In conclusion, MEN1-ZES can develop severe esophageal strictures, which can be treated by anti-acid therapy and repetitive endoscopic balloon dilation. Cautious stepwise procedures with intralesional corticosteroid injection facilitated safer esophageal dilation. Additional surgical resection of NET needs to be considered in severe refractory cases. The desirable extent of surgical resection is arguable in cases with multiple pancreaticoduodenal NETs, including functional ones.

## Abbreviations

CT: Computed tomography
MEN1: Multiple endocrine neoplasia
NET: Neuroendocrine tumor
PPI: Proton pump inhibitors
PTH: Parathyroid hormone
SASI: Selective arterial secretagogue injection
UGE: Upper gastrointestinal endoscopy
ZES: Zollinger-Ellison syndrome
